# THE EFFECT OF ILEOCECAL VALVE REMOVAL IN A MODEL OF SHORT BOWEL SYNDROME

**DOI:** 10.1590/0102-672020180001e1417

**Published:** 2019-01-07

**Authors:** Wangles Vasconcellos SOLER, Andre Dong LEE, Eugênia Machado Carneiro D’ALBUQUERQUE, Vera CAPELOZZI, Luiz Carneiro ALBUQUERQUE, Peretz CAPELHUCHNICK, Carmem Penteado LANCELOTTI, Flavio Henrique Ferreira GALVÃO

**Affiliations:** 1 Laboratory of Medical Investigations 37 - LIM 37, Faculty of Medicine, University of São Paulo; 2 Department of Surgery, Irmandade da Santa Casa de Misericórdia de São Paulo, São Paulo, SP, Brazil

**Keywords:** Short bowel syndrome, Protein malnutrition, Intestinal mucosa, Síndrome do intestino curto, Desnutrição proteico-calórica, Mucosa intestinal.

## Abstract

**Background:**

Short bowel syndrome is a harmful condition that needs experimental research.

**Aim:**

To assess the impact of the ileocecal valve removal in a model of short bowel syndrome, in order to investigate the evolution of the colon under this circumstance.

**Method:**

Fifteen Wistar rats were equitable divided into: Control (Sham), Group I (70% enterectomy preserving ileocecal valve) and Group II (70% enterectomy excluding ileocecal valve). After enterectomy was performed jejunoileal or jejunocecal anastomosis and sacrificed the animals on 30^th^ postoperative day for histomorphometric study of the colon. During this period, was observed the clinical evolution of the animals weekly including body weight measurement.

**Results:**

Group I and II presented progressive loss of weight. In Group I was observed diarrhea, perineal hyperemia and purple color of the colon during autopsy. Histomorphometry assay showed hypertrophy and hyperplasia of colon mucosa in Group I. In Group II the colon wall was thicker due to hypertrophy and muscular hyperplasia, and in mucosa vascular proliferation and inflammatory infiltrate were intense.

**Conclusion:**

This short bowel syndrome model is relevant and achieve 100% of survival. Animal’s weight loss was not altered by the presence or exclusion of the ileocecal valve. Animals with 70% of small bowel removal and presence of the ileocecal valve attained a better clinical evolution and histological colon adaptation than those without ileocecal valve.

## RESUMO

**Racional:**

Síndrome do intestino curto é condição clínica crítica e que precisa de pesquisa experimental.

**Objetivo:**

Avaliar o impacto da remoção da válvula ileocecal em um modelo de síndrome do intestino curto para investigar o comportamento do cólon nesta circunstância.

**Método:**

Quinze ratos Wistar foram divididos em três grupos de cinco: Controle (Sham), grupo I (enterectomia de 70% com preservação da válvula ileocecal), e grupo II (70% enterectomia de 70% excluindo a válvula ileocecal). Após a enterectomia foi restabelecido o trânsito com anastomose jejunoileal no grupo I e jejunocecal no grupo II. Os animais foram sacrificados no 30º dia do pós-operatório para histomorfometria do cólon. Durante este período, observou-se a evolução clínica semanal, incluindo a medição do peso corporal.

**Resultados:**

Grupos I e II apresentaram perda progressiva de peso. No grupo I houve diarreia, períneo hiperemiado e cor violácea do cólon durante a autópsia. A histomorfometria mostrou hipertrofia e hiperplasia da mucosa do cólon no grupo I. No grupo II a parede do cólon estava mais espessa devido à hipertrofia e hiperplasia das camadas muscular e mucosa onde a proliferação vascular e infiltração inflamatória foi intensa.

**Conclusão:**

Este modelo é factível e atingiu 100% de sobrevida. A perda de peso não foi alterada pela presença ou exclusão da válvula ileocecal. Animais com remoção de 70% do intestino delgado e presença da válvula ileocecal apresentaram melhor evolução clínica e adaptação histológica do cólon que os sem válvula ileocecal.

## INTRODUCTION

Short bowel syndrome occurs when more than 50% of the small intestine is resected. This condition causes malabsorption, abdominal pain, diarrhea, hydroelectrolyte disturbance, weight loss, malnutrition, dehydration, episodes of bacterial translocation, sepsis and death^4^ ^,^ ^22^. It´s most frequent causes in the pediatric group are necrotizing enteritis, intestinal atresia and small intestine volvulus; in adults inflammatory bowel disease, mesenteric ischemia and small intestine obstruction are more prevalent. In this situation, the mucosal surface of the remaining intestine may take up till two years to be adapted^2^ ^,^ ^22^.

 The best predictor of life expectancy after resection is the extension of the remaining intestine. In adults with small bowel remaining less than 50 cm, the probability of total parenteral nutrition dependence is 83% and in the pediatric group, the remaining extension of less than 10% of the intestinal length is predictive of permanent parenteral nutrition need^18^. The presence of the entire colon and ileocecal valve in the remaining intestine usually indicates a great possibility of intestinal adaptation and total parenteral nutrition weaning^10^. In large intestinal resections including the ileocecal valve, the time for oral diet full acceptance without complementation of parenteral nutrition is 18 months, while in the ones where the ileocecal valve was resected this period is 43 months^7^ ^,^ ^11^. These findings emphasize the importance of maintaining the ileocecal valve. 

The adaptive response in the context of large intestinal resections is basically characterized by enterocytes proliferation and villous hyperplasia, but many aspects of this process remains unclear, mainly the effect of the ileocecal valve^8^. 

The aim of this study was to analyze the effect of ileocecal valve removal after 70% small bowel resection in order to clarify the colonic pathological alterations and clinical aspects after this massive intestinal ressecion.

## METHODS

### Animals

Were used 15 male Wistar rats, weighing between 300-330 g from the vivarium of the Faculdade de Ciências Médicas, Santa Casa de São Paulo, São Paulo, SP, Brazil. The animals were fasted for 12 h prior to laparotomy. Rules of the International Council for Scientific Research for Laboratory Animals (International Council for Laboratory Animal Science) and the Council of Animal Care from the Institution (no. 223/97) were followed.

### Experimental design

Were randomly assigned three groups of five rats: Group I (GI), laparotomy, resection of 70% of the small intestine and jejunoileal end-to-end anastomosis; Group II (GII), laparotomy, resection of 70% of the small intestine and jejunocecum end-to-end anastomosis; Control group (Sham) - rats submitted to a simulation of the previous described procedure, performing a laparotomy and intestinal dissection like those described previously, but without intestinal resection and just an intestinal biopsy of the right colon in the antimesenteric wall that was closed by single stitches and the small bowel remained untouched. The animals were sacrificed on the 30^th^ postoperative day and removed the small and large intestine for histopathological study. During this period, was realized weekly evaluation of weight measurement and presence of diarrhea.

### Anesthesia and surgical procedures 

Anesthesia was performed by intraperitoneal use of ketamine hydrochloride (50 mg/kg) and chlorpromazine (50 mg/kg). All the animals underwent midline laparotomy, as follows:

 Group I - Intestinal length was measured by the antimesenteric border. Intestinal resection extended from 28 cm distal to the duodenojejunal angle (proximal resection margin) to 2 cm proximally to the ileocecal valve (distal margin of resection), corresponding to 70% of the intestine. Jejunoileal end-to-end anastomosis was then performed by extra-mucosal single-layer continuous 6-0 silk suture using 6 to 10 times magnificence. 

 Group II - The proximal margin of intestinal resection was the same as GI. The distal margin was also the same (2 cm proximal to the ileocecal valve), but the distal stump of the ileum was closed. The intestinal tract was reconstructed by an end-to-side anastomosis of the jejunum to the proximal cecum using extra-mucosal single-layer 6-0 silk continuous suture with 6 to 10 times magnificence.

Control Group (Sham) - Rats underwent right colon biopsy with 4 cm extension at 2 cm of the ileocecal transition. The colon wall was sutured by extra-mucosal single-layer 6-0 silk continuous suture using 6 to 10 times magnificence.

After operation, abdominal wall was closed in all groups using continuous 4-0 suture in the muscular and skin layers.

In the postoperative period, oral diet was initiated 24 h after surgery. Weight measurement and clinical evolution were performed weekly. In the 30^th^ postoperative day, animals were euthanized for autopsy. Was removed 1 cm of ascending colon 2 cm after ileocecal valve, opened it in the antimesenteric border and gently washed the lumen with 0.9% saline solution. The opened colon was secured in a cardboard and fixed in 10% formalin solution for subsequent staining by H&E staining. The histology sections were analyzed by two independents pathologists.

### Animals’ weight

All animals were weighted immediately before the operation and on 7^th^, 14^th^, 21^st^ and 30^th^ postoperative days.

### Macroscopic evaluation of the colon

It was performed in all animals at necropsy, especially considering the presence of color change and bowel dilatation.

### Histopathological analysis

The right colon was fixed in 10% formaldehyde for 24 h and 10 samples were taken per colon, two at each 0.3 cm intervals, from the ileocecal valve region. The material underwent routine cutting and inclusion techniques for conventional light microscopy. The three micrometers thickness sections were stained with H&E. Histological analyzes were performed under conventional light microscopy, with qualitative and quantitative studies.

 Qualitative study included the presence of inflammatory infiltrate and its components. For the quantitative study of histomorphometry was used a PZO K-15 drum eyepiece with a graduation of eight units, each unit corresponding to 20.28 micrometers in conventional Zeiss microscope, in the 100-fold increase. The following measures were performed: total wall thickness of the colon, thickness of the mucosa, height of the crypts and thickness of the muscular layer. The inflammatory infiltrate, containing macrophages, lymphocytes and plasmocytes, was graduated according to the following score: 0 corresponded to the absence of inflammatory cells; I to mild; II to moderate; III to intense infiltrate. 

## RESULTS

### Postoperative evolution

All the animals survived until the experiment end. All in GII presented periods of diarrhea starting from the 3^rd^ postoperative day and moderate hyperemia of the perineum at the end of the experiment. This finding was not observed in GI animals.

#### Weight variability

Animals from GI and GII presented progressive loss of weight that was significant from the 14^th^ postoperative day when compared with the ponderal evolution from Sham group. There was no statistical difference in weight loss between GI and GII animals at all analyzed times. At the end of the experiment, the median percentage of weight loss was 11% for GI and 14% for GII.

### Macroscopic assessment of the colon

**FIGURE 1:**
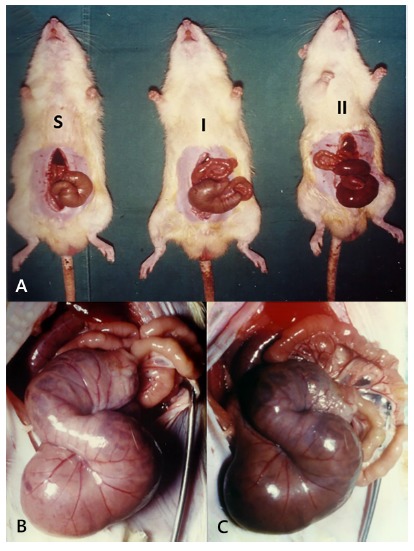
Macroscopic evaluation of colon: A) left to right: sham, group I and group II; B) cecum and colon dilatation; C) color change (purple appearance)

During necropsy Sham presented normal appearance (Figure 1A). Animals from GII showed important cecum and colon dilatation (Figure 1B) and color change (purple appearance - Figure 1C). In animals from GI, there was also colon enlargement but neither purple color appearance nor dilatation were noticed in the remaining colon (Figures 1B and C).

### Histopathological and morphometric analysis

**FIGURE 2:**
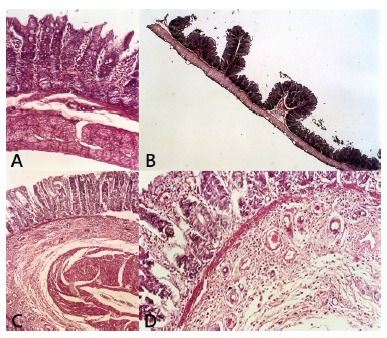
Segment of colon by H&E staining and visualized under optical microscope: A) control group showing uniform crypts lined by goblet cells and supported by prominent lamina propria infiltrated by a small number of lymphomonuclear cells and the submucosa is continued with regular fibers of muscularis mucosae and muscularis externa; B) GI showing well-formed glands and crypts lined by epithelial-goblet cells, with scant lamina propria, delayed shedding of otherwise normal surface epithelial cells leading to infolding of the crowded epithelial cells, creating a serrated epithelial profile; C and D) GII showing remodeling of the submucosa by prominent angiogenesis and lymphomonuclear cells infiltration, hypertrophy and hyperplasia of both circular and longitudinal muscle (magnification: Ax100; Bx40; Cx100; Dx400).

Histopathological analysis showed minimum inflammatory infiltrate in animals of Sham and GI and pronounced vascular proliferation, inflammatory infiltrate and increased wall thickness of the colon in animals of GII. Iinflammatory infiltrate in all groups was composed by macrophages, plasmocytes and rare lymphocytes. In the morphometric analysis, was observed normal appearance of colon layers in Sham group (Figure 2A), and a statistically significant increase in total wall thickness of the colon in GI and II, due to hypertrophy and hyperplasia (Tables 1 and 2). Interestingly, there was a different pattern in colon wall morphometry. In animals from GI there were a significant predominance of mucosal tunic and height of the crypts enlargement, in which hyperplastic polyps in the mucosa were a frequent finding (Figure 2B). In GII, both muscular and mucosa layers were enlarged with predominance of muscular layer (Figure 2C and 2D, Table 3). Qualitative analysis of infiltrade in mucosa and submucosa of the three groups presented lymphocytes, macrophages and plasmocytes with greater intensity in GII (Table 4).

**TABLE 1:** Statistical analysis of the summative evaluation of the total thickness of the large intestine

Animal	Control group		Group I		Group II	
1	å1-10	3,21	å1-10	4,45	å1-10	5,52
2	å1-10	3,03	å1-10	3,86	å1-10	6,00
3	å1-10	3,36	å1-10	3,86	å1-10	8,29
4	å1-10	2,23	å1-10	2,45	å1-10	3,22
5	å1-10	3,36	å1-10	2,44	å1-10	5,54
x	åx1-x5	3,04	å x1-x5	3,41	å x1-x5	5,71
dn	0,47		0,91		1,80	
Contrasts (R1 - RJ)			Significance			
G1 X Gc			p>0,05			
G2 X Gc			p=0,05			
G1 X G2			p>0,05			

Test non-parametric Kruskall-Wallis H of=7.01; critical value=5.99; 1 unit=28.20 mm; S 1-10=sum of the measures of the samples; X = average of the Sommatives; dn=standard deviation of the samples

**TABLE 2:** Statistical analysis of the summative measurements of thickness of the large intestine mucous layer

Animal	Control group		Group I		Group II	
1	å1-10	2,33	å1-10	2,53	å1-10	2,69
2	å1-10	1,73	å1-10	2,90	å1-10	4,04
3	å1-10	2,70	å1-10	3,60	å1-10	4,53
4	å1-10	2,53	å1-10	3,00	å1-10	6,53
5	å1-10	2,34	å1-10	3,22	å1-10	5,75
x	åx1-x5	2,63	å x1-x5	2,67	å x1-x5	4,46
dn	0,32	0,35		1,23		
Contrasts (R1 - RJ)			Significance			
G1 X Gc			p=0,05			
G2 X Gc			p>0,05			
G1 X G2			p=0,05			

Kruskall-Wallis non-parametric testing H=7.01; critical value=5.99; 1 unit=28,20 **mm;** å1-10=sum of the measures of the samples; X=mean of biopsy measurements; dn =standard deviation

**TABLE 3:** Statistical analysis of the thickness of the muscular layer of the large intestine and comparison between different groups

Animal	Control group		Group I		Group II	
1	å1-10	0,72	å1-10	0,88	å1-10	0,69
2	å1-10	0,42	å1-10	0,76	å1-10	1,33
3	å1-10	0,51	å1-10	0,72	å1-10	1,23
4	å1-10	0,58	å1-10	0,59	å1-10	2,00
5	å1-10	0,51	å1-10	0,52	å1-10	1,51
x	å x1-x5	0,54	å x1-x5	0,69	å x1-x5	1,39
dn	0,09	0,12		0,42		
Contrasts (R1 - RJ)			Significance			
G1 X Gc			p=0,05			
G2 X Gc			p>0,05			
G1 X G2			p>0,05			

Kruskall-Wallis non-parametric testing H=7.01; critical value=5.99; 1 unit=28.20 mm; S 1-10=sum of the biopsies; X=average of the biopsy; dn=standard deviation

**TABLE 4:** Qualitative study of inflammatory infiltrate in different groups of animals, according to cell type and weight scale of intensity of the inflammatory infiltrate

Cell type	Control group	Group I	Group II
Macrophages	2	2	3
Lymphocytes	1	1	1
Plasmocytes	2	1	3

Intensity of inflammatory infiltrate: 0=absence of undercover; 1=rare presence of cells; 2=frequent meeting of cells; 3=intense undercover

## DISCUSSION

To the best of our knowledge, this is the first survey assessing 70% of small bowel resection with and without ileocecal valve. Was chosen 70% based on a previous pilot study in which was found a high rate of discomfort and mortality of the rats with resection of 90%. In this model, weight loss was more related to the length of the removed small bowel rather than the exclusion of the ileocecal valve. Also, all animals survive until experimental end, making this model relevant to study long-term effect of short bowel syndrome and to create translational experiments of new intestinal transplantation models^3^ ^,^ ^5^ ^,^ ^6^ ^,^ ^21^using rats recipients with short bowel syndrome, like in clinical management. 

Barra et al.^2^ studied the consequences of ileocecal valve resection without enterectomy in intestinal microbiota. There was alteration of the intestinal microbiota only in resected ileocecal valve group, that was greater in the terminal ileum than in the colon.In this study, was found an increase in the thickness and inflammatory infiltrate in the colon of GII animals. The microbiota alterations demonstrated by Barra et al.^2^may be correlated to this finding. Saad et al.^16^ observed that extended intestinal resection did not determine significant weight loss after 60 postoperative days compared to control. The current experiment had a 30-day observation period with a continuous weight loss. It is reasonable to assume that we could find weight gain or stabilization tendency if the period of observation would be longer than 30 days.It is understandable that the resection of 70% of the small bowel results in better intestinal absorption in the 30% of the preserved area; this didn’t happen in the study in which 90% of the small bowel was resected. Besides, in the resection of 70% of small intestine, theilealpapilla seems to improve intestinal function, with no diarrhea, differently from the group without preservation of the ileocecal valve.

Miloneet al.^12^ using different extensions of intestinal resection, observed that the colon actively participating in the adaptive response of the small bowel.We also observed an important adaptation of the colon mucosa, mainly when the ileocecal valve was preserved. This morphological change may be related to the readaptation of the colon assuming an absorptive function like small intestine.

Obertopet al.^14^ in a rat model of resection of the proximal one-third of the small bowel (jejunectomy) observed early manifestations of cell proliferation on the 2^nd^ day with augmentation of absolute amounts of RNA and DNA in the distal small bowel and colon that was persistent in midgut tending to be less intense in distal segments. The hyperplasia reachhigherintensity in the ileum on the second postoperative week. In our experiment, we observed sign of hyperplasia of the mucosa in the colon just with the extensive small intestine resection, mainly when the ileocecal valve is preserved.

The morphological and functional repercussions found in experimental surgery were confirmed in clinical studies. Thompson^20^, studying large intestinal resections, noted that short bowel syndrome depends on some factors like: length of the resected intestine, resected segment (jejunum or ileum), underlying intestinal disease, presence or absence of the ileocecal valve, and functional status of the other organsof the digestive system. Rasslan et al.^15^ observed malnutrition and diarrhea difficult to control in the early postoperative period of patients submitted to 75% of the small intestine resection. The jejunal resections were better tolerated, presenting slower intestinal transit, while patients with ileal resections got faster intestinal transit and steatorrhea. When the ileocecal valve was resected, they observed a higher hydroelectrolytic loss by the stoma and greater difficulty in adapting to the diet after feeding. This clinical study supports the results of our experiment of the effect of ileocecal valve resection. Gouletet al.^7^ studying pediatric patients with approximately 40 cm of small bowel remaining, compared those that maintained the ileocecal valve with the ones whoseileocecalvalve was removed and concluded that the inclusion ornot of the ileocecal valve had no influence in the mortality. In our experiment, there was also no difference in mortality and body weight loss between the groups with and without valve.

The colon reacts actively in the adaptive response of extensiveenterectomy. Nundyet al.^13^ demonstrated that the increase of the colon in largeileal resection results in hyperplasia since the 7^th^postoperative day, increasing to the maximum around the 30^th^postoperative day, maintaining a plateau up to the 6^th^month of observation.In our experiment, it was also observed hyperplasia on 30^th^postoperative day. 

Using scanning electron microscopy, Tamames Gomez et al.^19^ observed that the colon mucosa ofenterectomized rats had at 60 postoperative days protrusions with follicular aspectlike the mucosa of the small intestine. Miloneet al.^12^ reported similar alterations until the 5^th^postoperative month, when the alterations were stabilized. We also observed these alterations in histometry. These histopathological alterations cause avicarious stimulus in the remaining colon due to the compensatory mechanism, trying to supply the metabolic needs created by the extensive intestinal resection. 

The enlarged wall thickness of the remaining colon after extensive small bowel resection is caused by an increase in all its components. As it can be seen in statistic tables the exclusion of ileocecal valve is the main cause of significant improvement of the mucosa and muscular layer in comparison between GII e control group. Further research must be follow in order to explain if the intestinal of remnant small bowell content in the absence of ileocecal valve has a role in this response. This response, like that observed in the remaining small intestine^16^, indicates an adaptive response that probably involves all digestive tract in largeenterectomies. In this experiment, was observed intense vascular proliferation in the chorion, severalmast cells, and an importantconjunctive proliferationin GII. This aspect of theinfiltrateand other changes are like those stated by Allen^1^ in the colon of patients who undergone ischemia. We attributed the reddish color of the colon to the intense cellular hyperplasia of the various layers and the relative ischemia, related to an intense cellular proliferation not accompanied by corresponding vascularization.This finding was also observed by Milone et al.^12^ and Tamames Gomes et al.^19^.

## CONCLUSIONS

The present short bowel syndrome model is relevant and achieve 100% of survival. Animal’s weight loss was not altered by the presence or exclusion of the ileocecal valve. Animals with 70% of small bowel removal and presence of the ileocecal valve attained a better clinical evolution and histological colon adaptation than those without it.
